# Assessments of Arterial Stiffness and Endothelial Function Using Pulse Wave Analysis

**DOI:** 10.1155/2012/903107

**Published:** 2012-05-14

**Authors:** Lee Stoner, Joanna M. Young, Simon Fryer

**Affiliations:** ^1^School of Sport and Exercise, Massey University, P.O. Box 756, Wellington 6140, New Zealand; ^2^Lipid and Diabetes Research Group, Diabetes Research Institute, Christchurch Hospital, Christchurch 8011, New Zealand; ^3^Department of Medicine, University of Otago, Christchurch 8140, New Zealand; ^4^School of Sciences and Physical Education, University of Canterbury, Christchurch 8140, New Zealand

## Abstract

Conventionally, the assessments of endothelial function and arterial stiffness require different sets of equipment, making the inclusion of both tests impractical for clinical and epidemiological studies. Pulse wave analysis (PWA) provides useful information regarding the mechanical properties of the arterial tree and can also be used to assess endothelial function. PWA is a simple, valid, reliable, and inexpensive technique, offering great clinical and epidemiological potential. The current paper will outline how to measure arterial stiffness and endothelial function using this technique and include discussion of validity and reliability.

## 1. Introduction

Cardiovascular disease (CVD), the leading cause of mortality in the Western world [1], has a very long asymptomatic phase of development, starting as early as the first decade of life [2]. It is imperative, therefore, that clinical scientists and epidemiologists have at their disposal simple, valid, and reliable techniques to assess and track the progression of CVD. Noninvasive assessment techniques fall under two broad categories: those that assess endothelial health and those that assess arterial stiffness. Assessment of endothelial function indicates the functional health of the vascular system, whereas arterial stiffness assesses structural characteristics. Together, these techniques may provide complimentary indices of CVD risk.

Conventionally, assessments of endothelial function and arterial stiffness require different sets of equipment, making the inclusion of both tests impractical for clinical and epidemiological studies. Pulse wave analysis (PWA) is a simple and noninvasive technique that has been widely used in epidemiological [3] and interventional studies [4]. PWA provides useful information regarding the mechanical properties of the arterial tree and the ventricular-vascular interaction [5] and can also be used to assess endothelial function [6]. PWA is a simple, valid, reliable, and inexpensive technique, offering great clinical and epidemiological potential. The current review will outline how to measure arterial stiffness and endothelial function using this technique and include discussion of validity and reliability.

## 2. Arterial Stiffness

Arterial stiffness is a general term that collectively describes distensiblility, compliance, and elastic modulus of the arterial vascular system. These properties are not homogenous along the arterial tree and muscular and elastic vessels differ. Arterial stiffness can be measured systemically, regionally, or locally. Local measurements provide important physiological information and are more quantitative and sensitive than systemic indices. However, these measurements give no indication of how the artery of interest interacts with central function (i.e., the heart) as part of an integrative system. Regional arterial stiffness is measured at arterial sites of major physiologic importance such as the aorta where the arterial buffering function is principally expressed, or a particular limb. Systemic arterial stiffness affects the global buffering properties of the arterial system, just as arterial blood pressure can be considered as a global value of hemodynamic load, systemic arterial stiffness reflects the overall opposition of large arteries to the pulsatile effects of ventricular ejection.

A number of methodologies have been applied to the *in vivo* assessment of arterial stiffness. These methodologies fall into three broad groups: (1) relating change in area of an artery to distending pressure, that is, local arterial stiffness, (2) measuring pulse wave velocity, that is, regional arterial stiffness, and (3) pulse wave analysis, that is, systemic arterial stiffness. Ultrasound and magnetic resonance imaging (MRI) are capable of measuring local arterial stiffness [21–23] as well as pulse wave velocity [24–30], but these methodologies require expensive equipment (especially in the case of MRI) and a high level of technical expertise and are often impractical within the clinical or epidemiological setting. PWV can also be assessed using dedicated equipment, including oscillometric [31–34], tonometric [32, 35–37], volume plethysmographic [38, 39], and photo plethysmographic [40–43] devices. These devices either measure the pulse wave at two peripheral sites or record the electrocardiogram and measure the pulse wave at a peripheral site, to estimate the regional arterial stiffness. Alternatively, a number of devices are also available to estimate *systemic* arterial stiffness using PWA.

### 2.1. Measurement

A number of commercial devices are available to automate PWA assessments, including Compilor (tonometric device) [44, 45], Sphygmocor (tonometric device) [44, 46–49], PulsePen (tonometric device) [50, 51], ARCSolver [47, 52] (oscillometric device), Arteriograph [33, 53–57] (oscillometric device), Omron (oscillometric device) [55, 58], PulseCore [59, 60] (oscillometric device), Viscorder [32, 34, 49] (oscillometric device), and PulseTrace [6, 42, 48, 61] (photoplethysmographic device). Applanation tonometry is considered the “gold standard” and is the most widely used technique [62]. A probe is conventionally placed on the skin overlying the radial artery, and pressure is applied to distort or applanate (flatten) the artery, creating a signal which approximates arterial pressure. The peak and trough of the radial pulse wave correspond, respectively, to systolic and diastolic blood pressure measured conventionally on the brachial artery, since blood pressure is practically identical in brachial and radial arteries [63]. Mean blood pressure is determined by integration of the radial wave. A generalized transfer factor is then used to generate the corresponding central arterial waveform [64–67].

**Figure 1:**
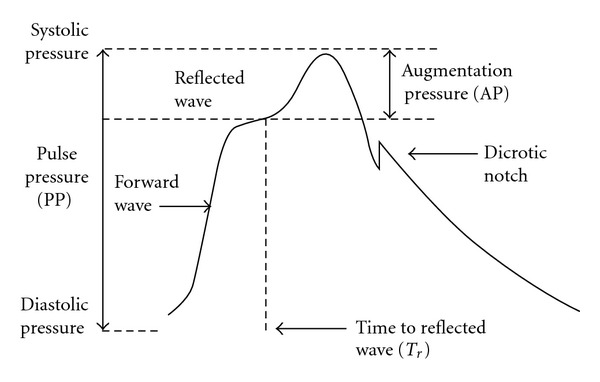
Aortic pulse pressure waveform. Systolic and diastolic pressures are the peak and trough of the waveform. Augmentation pressure is the additional pressure added to the forward wave by the reflected wave. Augmentation index is defined as the augmentation pressure as a percentage of pulse pressure. The dicrotic notch represents closure of the aortic valve and is used to calculate ejection duration. Time to reflection is calculated as the time at the onset of the ejected pulse waveform to the onset of the reflected wave.

Figure 1 shows typical features of the aortic pulse pressure waveform, from which can be derived augmentation pressure (AP), augmentation index (AIx), and arrival time of reflected waves at the central aorta (*T*r). *T*r represents the time from the onset of the ejected pulse waveform to the onset of the reflected wave and reflects aortic pulse wave velocity [68]. AP is the additional aortic systolic pressure generated by the return of the reflected waves at the central aorta, expressed in absolute terms [62]. AIx is the AP as a percentage of central pulse pressure and is a composite measure of aortic wave reflection and *systemic arterial stiffness* [62, 69]. Although the timing of the arrival of the reflected wave at the proximal aorta is largely determined by large artery PWV, AIx is not interchangeable with PWV. It is influenced by vasoactive drugs independently of PWV [70], suggesting that it is also determined by the intensity of wave reflection, which, in turn, is determined by the diameter and elasticity of small arteries and arterioles. A number of variables are known to influence AIx. AIx increases with MAP [71] and is inversely related to body height [72] and heart rate [73, 74], with a 10 bpm increase in heart rate resulting in a 4% reduction in AIx [73]. AIx should be normalized for a heart rate of 75 beats per minute (AIx@HR75). One of the most widely used devices, SphygmoCor, automatically adjusts the AIx at an inverse rate of 4.8% for each 10 bpm increment. The AIx@HR75 is only calculated when the patient's heart rate is between 40 and 110 bpm. Outside of this range the software will display an N/C indicating that no calculation was possible.

**Figure 2:**
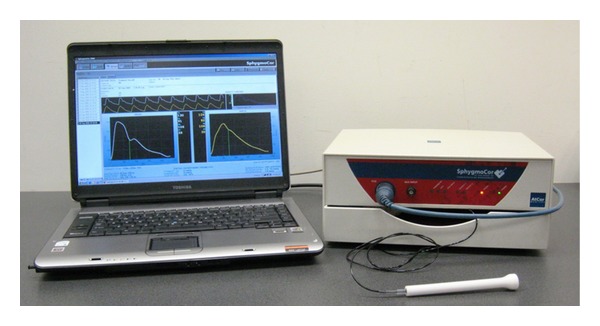
Electronics module (SphygmoCor device, AtCor Medical, Sydney, Australia).

**Figure 3:**
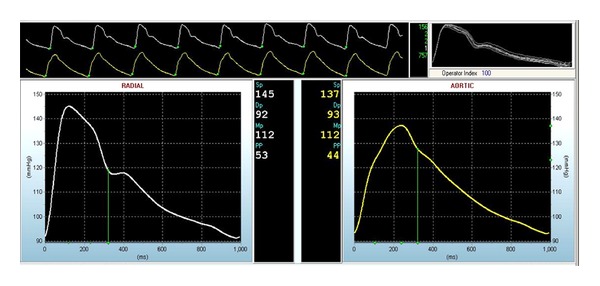
Radial artery applanation tonometry recording. The upper long panel shows the radial pressure waveform above the derived central pressure waveform. The upper right panel shows the overlaid radial waveforms, including the operator index, and the middle panel shows the quality control indices. The bottom left panel demonstrates a magnified radial arterial waveform. Systolic and diastolic pressures are 145/92 mmHg. The bottom right panel provides a magnified derived central pressure waveform. Central pressure is 137/93 mmHg.

Using the SphygmoCor to illustrate the procedure (a typical setup is shown in Figure 2), PWA takes approximately 20 minutes to complete. Following at least 10 minutes supine rest, brachial artery systolic and diastolic blood pressures are measured in the nondominant arm and used to calibrate the PWA measurements taken on the radial artery. Radial artery waveforms are then recorded in duplicate. A high-fidelity tonometer is used to obtain pressure waveforms by applying gentle pressure over the nondominant radial artery and repositioning the device until the greatest pulse signal is detected. Data is collected directly into a personal computer, and recordings are assessed visually to ensure that the best possible recording is obtained. After 20 sequential waveforms are acquired, an averaged peripheral waveform is generated and a corresponding aortic waveform is derived (see Figure 3). When consecutive AIx@HR75 readings differ by more than 4%, a third reading is obtained, and the mean of the closest two readings is taken.

### 2.2. Validity

There is evidence that increased aortic wave reflections have adverse effects on ventricular afterload and coronary perfusion, and their pathological role has been demonstrated in several diseases [75–78]. Furthermore, increased central arterial wave reflections have been shown to independently predict cardiovascular risk and mortality [5, 79, 80]. Increased amplitude and the earlier return of the reflected wave within the cardiac cycle augments the central systolic blood pressure, resulting in increased wave reflections [69]. The amplitude and timing of reflected pressure waves are determined primarily by vascular elasticity, peripheral vascular resistance, heart rate, and left ventricle function [81].

### 2.3. Reliability

Any valid technique utilised for the measurement of physiological variables must be reproducible [82]. A high intra- and interobserver reproducibility of baseline AIx has been observed in healthy controls and patients with cardiovascular disease and renal dysfunction [7, 38, 82–94]. Good reproducibility of baseline time to reflection (*T*r), an alternative index to AIx, has been reported in several studies [88, 91, 94]. However, the majority of these trials examined the reproducibility using simple Bland-Altman analysis, whereas studies using more definitive intra-class correlation coefficient (ICC) and the contribution of variance components are limited [81, 82, 91, 93, 94]. ICC values for repeated measurements taken at hourly or weekly intervals have been reported to be 0.72–0.90 for AIx [91, 94], 0.90 for AP [81], and 0.43–0.84 for *T*r [81, 91, 94]. In addition, few trials have reported the reproducibility of heart rate corrected AIx [82, 91, 93], although it is frequently used in wave reflection studies. While this technique potentially offers a valid and reliable marker of CVD risk, further study is required to determine sample size recommendations for AIx normalized to heart rate.

### 2.4. Recommendations

The PWA technique is particularly suitable for incorporation into clinical trials. Strengths of this technique include simplicity of assessment, relatively short training requirements for investigators, low time commitment for subjects, noninvasiveness, portability, and cost-effectiveness. Both AIx and AP confer similar reproducibility and have been reported as more reliable than *T*r. Since AIx is strongly influenced by heart rate, both AIx and AP should be corrected for this confounding factor [73]. Further study is warranted to determine whether the three indices of arterial stiffness provide additive prognostic value.

## 3. Endothelial Health

Functionally, the endothelium is a large autocrine, paracrine, and endocrine organ that plays a key role in vascular homeostasis [95]. Endothelial dysfunction is a pivotal, yet potentially reversible, step that has been shown to precede and predict overt CVD [96]. The endothelium has been recognized for the important role it plays in regulating vascular reactivity via the release of dilator mediators, including nitric oxide (NO) [97–100], prostaglandins [101], and endothelial-derived hyperpolarizing factor [102, 103]. The capacity of the endothelium to regulate vascular tone (reactivity) is used to confirm the health of the endothelium.

Established methodologies for evaluating peripheral endothelial function include strain-gauge venous occlusion forearm plethysmography [104, 105], ultrasound-measured flow-mediated dilation (FMD) [105–107], peripheral arterial tonometry (e.g., using the EndoPat device) [105], and laser Doppler flowmetry [105]. These techniques assess the vasodilator responses to endothelium-dependent stimuli such as acetylcholine and increased shear stress and to endothelium-independent stimuli, including sodium nitroprusside and glyceryl trinitrate (GTN). The two most commonly used techniques are strain-gauge plethysmography and FMD, with FMD being considered the “gold standard” for assessing endothelial function. FMD is a noninvasive, valid [10–13], and moderately reliable [14, 15] technique but is expensive and highly technical (Table 1). Strain-gauge plethysmography also offers acceptable reliability [17–20] but is an invasive technique when coupled with intra-arterial infusion of vasodilators (Table 1). As such, these techniques are often impractical for use in clinical trials or epidemiological studies. Alternatively, a limited number of studies have used PWA to assess endothelial function.

### 3.1. Measurement

Endothelial function can be assessed noninvasively by evaluating the effects of inhaled salbutamol on the AIx [6–8]. AIx is a measure of systemic arterial stiffness [62]. Notably, NO, considered the central molecule governing endothelial function [97–100], is a key modulator of arterial stiffness [108]. Chowienczyk et al. [6] demonstrated that salbutamol, a *β*2 agonist and endothelium-dependent vasodilator, in part reduces wave reflection by activation of the L-arginine-NO pathway.

The test takes approximately 35 minutes to complete; following at least 10 minutes supine rest, baseline PWA is recorded as described above, and PWA recordings are then repeated after 5, 10, 15, and 20 minutes after the administration of 400 *μ*g inhaled salbutamol. The maximal decrease in AIx from baseline following a salbutamol challenge is used as an index of endothelial function. Endothelium-independent function can also be assessed using PWA by measurement of the reduction in AIx following sublingual GTN administration.

### 3.2. Validity

Wilkinson et al. [7] and Hayward et al. [8] evaluated the effects of inhaled salbutamol and sublingual GTN on the AIx. Investigators observed a significant correlation between the salbutamol-mediated reductions in AIx and increase in forearm blood flow during infusion of acetylcholine that was abolished by coadministration of NG-monomethyl-L-arginine (L-NMMA), an endothelial NO synthase inhibitor, suggesting that this may represent a valid approach for assessing endothelial function. Conversely, coadministration of intravenous L-NMMA has no influence on sublingual GTN-mediated reductions in AIx, consistent with an endothelium-independent effect [37, 74]. To date, relatively few clinical studies have employed PWA to assess endothelial function. However, preliminary validation studies have demonstrated blunted AIx responses to salbutamol in subjects exhibiting diabetes [6], hypercholesterolaemia [7], coronary artery disease [8], and peripheral vascular disease [9] among others. 

### 3.3. Reliability

Studies examining the reproducibility of salbutamol-mediated effects on AIx are limited [7, 8, 82]. Hayward et al. [8] assessed reliability in healthy subjects using Bland-Altman analysis and reported excellent between-day mean difference (*d* = 0.9 ± 2%). Wilkinson et al. [7] also assessed reliability in healthy subjects using Bland-Altman analysis and reported similar findings; between-day mean difference in the AIx response was −2.3 ± 3.0 for salbutamol-mediated changes and 0.2 ± 6.5 for glyceryl trinitrate (GTN)-mediated changes. More recently, Paul et al. [82] produced discrepant findings when they assessed within-day reliability using the more definitive ICC method [109]. Relatively low reliability was reported for control (ICC: 0.18) and chronic heart failure groups (CHF) (ICC: 0.04). However, relatively low reliability was also reported for GTN-mediated changes in AIx for both control (ICC: 0.58) and CHF groups (ICC: 0.17). Endothelial function assessments were repeated at hourly intervals, and the authors suggest that the low ICC values may reflect diurnal variability or a carryover effect from the first assessment. Further study is warranted to determine the reliability and sample size requirements for this promising technique.

### 3.4. Recommendations

Salbutamol-mediated effects on AIx can be used to estimate endothelial function. This technique offers a number of advantages, namely simplicity of assessment, relatively short training requirements for investigators, low time requirement, portability, cost-effectiveness, ease of use, good reliability indicated by preliminary studies, and the salbutamol challenge isolating the NO pathway. However, more clinical studies are required to further validate this test. Also, while NO is unarguably an important molecule governing endothelial function, the endothelium can release additional complimentary or compensatory molecules [110], most notably prostacyclin [101] and endothelial-derived hyperpolarizing factor [102, 103]. The relative importance of these molecules varies by vascular bed and between individuals, particularly between individuals exhibiting a number of diseased states [111–120]. Therefore, while this test can provide a snapshot of endothelial function it may not provide a complete picture. Further study is warranted to determine whether baseline AIx (systemic arterial stiffness) and AIx following salbutamol inhalation confer additive prognostic value. Further study is also required to clarify sample size recommendations for this test.

## 4. Conclusion

PWA is a simple technique capable of assessing systemic arterial stiffness and endothelial function. This test is particularly suitable for clinical and epidemiological studies. Further study is required to determine: (1) sample size requirements, and (2) whether baseline AIx (systemic arterial stiffness) and AIx following salbutamol inhalation confer additive prognostic value.

## Figures and Tables

**Table 1:** Comparison of noninvasive techniques for assessing endothelial function.

Technique	Equipment	Cost	Skill level	Test time	Validity	Reliability	Ref
PWA	Applanation tonometry	Medium US$15,000	Low	35 mins	Medium*	Medium-high* *d *= 0.9–2.3%	[6–9]
FMD	(i) Ultrasound (ii) Tourniquet	High >US$50,000	High	20 mins	High	Medium *CV*: 14–50%	[10–16]
Plethysmography	Strain-gauge	Low US$10,000	Med.	30 mins	Medium	Medium *CV: *8–27%	[17–20]

FMD: flow-mediated dilation; CV: coefficient of variation; *d*: sample bias (mean difference); PWA: pulse wave analysis. *Further study is needed to corroborate these findings.
